# Supplementary figures and images for: Size–curvature constraint in the closing motion of Venus flytrap leaves
Source: PLoS One. 2026 May 26;21(5):e0349246. doi: 10.1371/journal.pone.0349246 (PMC13210226; doi:10.1371/journal.pone.0349246)

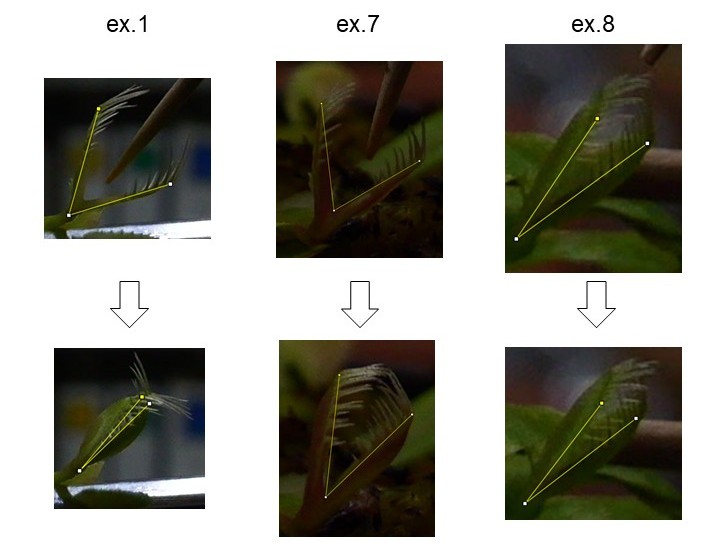

Supplement: S1 Fig — The opening angle was measured as the angle of the lines connecting the leaf joint and the base of the teeth. (JPG) [file pone.0349246.s001.jpg]

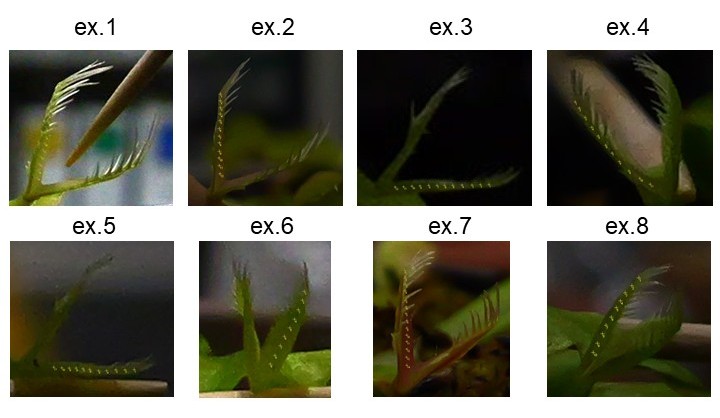

Supplement: S2 Fig — Yellow points indicate points detected along the midline. (JPG) [file pone.0349246.s002.jpg]

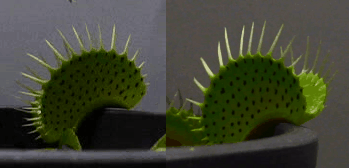

Supplement: S1 Movie — (GIF) [file pone.0349246.s003.gif]
