# Supplementary material for: Size–curvature constraint in the closing motion of Venus flytrap leaves
Source: PLoS One. 2026 May 26;21(5):e0349246. doi: 10.1371/journal.pone.0349246 (PMC13210226; doi:10.1371/journal.pone.0349246)
Supplement: Attachment — Submitted filename: review letter.docx [file pone.0349246.s004.docx]

Authors completed the 3D morphology reconstruction of the Venus flytrap by micro-CT scanning, and deduced a non-dimensional parameter by the geometric value obtained above to feature the motion of the Venus flytrap. The main conclusion is that the non-dimensional index they deduced is correlated to the angular velocity of the snapping. The verification of the relationship between the geometric parameters and dynamic motion is essential for the research of the Venus flytrap. Concerning the novelty and originality, I suggest to accept this paper after minor revision. However, there are several questions I hope to receive response from authors:

1. Page 2, line 45: actually, there is one paper published mentioned that only one trigger could initiate the motion of the plant. For your reference: *A single touch can provide sufficient mechanical stimulation to trigger Venus flytrap closure*.
2. Page 3, line 103: the non-dimensional parameter α was proposed by Yeol et al., could you explain why you choose this parameter and what new based on your analysis comparing their previous work?
3. Page 4, line 131-132; Page 5, line 169-170: could you explain the value difference of the D, because you mentioned that D for open is 2.6mm, and close is 0mm is page 4, and then D for open is 0.0, and close is 0.7? Besides, please describe the reason this value equals zero.
4. Page 5, line 165-166: It looks like that the closing time is pretty long, why this happen? Did you trigger the motion on site, or you have to cut the leaf for imaging?
5. Page 6, line 181-183: You used two velocity parameters to describe the motion, which should be better to represent the natural notion of the plant?
6. Page 6, line 198-199: Could you verify the two-layer model by your 3D CT data?
