# Supplementary material for: Size–curvature constraint in the closing motion of Venus flytrap leaves
Source: PLoS One. 2026 May 26;21(5):e0349246. doi: 10.1371/journal.pone.0349246 (PMC13210226; doi:10.1371/journal.pone.0349246)
Supplement: Attachment — Submitted filename: review letter_round2.docx [file pone.0349246.s006.docx]

The manuscript presents a clear and well-structured study combining micro–CT imaging, 3D reconstruction, and geometric modeling to investigate the closing motion of Venus flytrap leaves. The identification of a size–curvature relationship is interesting and potentially valuable, particularly for the design of bioinspired soft structures.

Overall, the work is carefully conducted, and the revisions have improved the manuscript. I find the study suitable for publication after minor clarifications that would further enhance readability and consistency.

**1. Clarification and consistency of parameter D (important)**

In the original geometric formulation proposed by Poppinga and Joyeux, the parameter **D** is defined as a *geometric quantity representing the maximum separation between the two lobes in the reference (closed) configuration*, and is used to construct a lifelike initial shape of the trap.Importantly, in that framework, D is treated as a **fixed geometric parameter**, rather than a dynamic variable describing the temporal evolution of the motion.

In this paper, the parameter D plays a central role throughout the manuscript, as it is used:

1. to distinguish open and closed states (e.g., D = 0 vs D > 0),

2. to describe the deformation process over time,

3. to construct the size–curvature relationship (e.g., D–H morpho space).

Given this central importance, the definition and interpretation of D should be as precise and consistent as possible. At present, D appears to serve multiple roles: a geometric parameter in the surface model (Eq. 1), a descriptor of deformation state, and, in the discussion, a proxy for underlying physiological processes.

While these interpretations are individually reasonable, their combination can be somewhat ambiguous. Please clearly define D in a single, primary sense (e.g., geometric descriptor vs. state variable), and explicitly state how the other interpretations relate to this definition (e.g., whether they are conceptual or derived).

Even a short clarifying paragraph would greatly improve the conceptual clarity of the manuscript.

**2. Physical interpretation of D**

The added physiological interpretation of D is interesting and provides useful intuition. However, because D is introduced through a geometric model and extracted from morphological fitting, its connection to quantities such as: differential strain, turgor pressure, or ion-mediated processes remains indirect.

To avoid overinterpretation, I suggest: explicitly framing this connection as qualitative or interpretive, unless a direct quantitative relationship is established. This will strengthen the rigor of the manuscript while preserving its interdisciplinary perspective.

**Conclusion**

This is a well-executed and interesting study that provides a useful geometric perspective on Venus flytrap mechanics. The manuscript is suitable for publication after minor revisions addressing the points above.
